# Identification of a Novel miR-195-5p/PNN Axis in Colorectal Cancer

**DOI:** 10.3390/ijms25115980

**Published:** 2024-05-30

**Authors:** Emanuele Piccinno, Viviana Scalavino, Nicoletta Labarile, Lucia De Marinis, Raffaele Armentano, Gianluigi Giannelli, Grazia Serino

**Affiliations:** National Institute of Gastroenterology S. De Bellis, IRCCS Research Hospital, Via Turi 27, 70013 Castellana Grotte, BA, Italy; emanuele.piccinno@irccsdebellis.it (E.P.); viviana.scalavino@irccsdebellis.it (V.S.); nicoletta.labarile@irccsdebellis.it (N.L.); lucia.demarinis@irccsdebellis.it (L.D.M.); raffaele.armentano@irccsdebellis.it (R.A.); gianluigi.giannelli@irccsdebellis.it (G.G.)

**Keywords:** microRNA, CRC, PNN, miR-195-5p, desmosome, keratins, cell adhesion, cell junctions

## Abstract

Pinin (PNN) is a desmosome-associated protein that reinforces the organization of keratin intermediate filaments and stabilizes the anchoring of the cytoskeleton network to the lateral surface of the plasma membrane. The aberrant expression of PNN affects the strength of cell adhesion as well as modifies the intracellular signal transduction pathways leading to the onset of CRC. In our previous studies, we characterized the role of miR-195-5p in the regulation of desmosome junctions and in CRC progression. Here, with the aim of investigating additional mechanisms related to the desmosome complex, we identified PNN as a miR-195-5p putative target. Using a public data repository, we found that PNN was a negative prognostic factor and was overexpressed in colon cancer tissues from stage 1 of the disease. Then, we assessed PNN expression in CRC tissue specimens, confirming the overexpression of PNN in tumor sections. The increase in intracellular levels of miR-195-5p revealed a significant decrease in PNN at the mRNA and protein levels. As a consequence of PNN regulation by miR-195-5p, the expression of KRT8 and KRT19, closely connected to PNN, was affected. Finally, we investigated the in vivo effect of miR-195-5p on PNN expression in the colon of AOM/DSS-treated mice. In conclusion, we have revealed a new mechanism driven by miR-195-5p in the regulation of desmosome components, suggesting a potential pharmacological target for CRC therapy.

## 1. Introduction

Cell–cell adhesion and interactions play a key role in several biological processes and in the establishment of intercellular junctional complexes, such as desmosomes, adherens junctions, and tight junctions [1,2]. An understanding of cellular activity and tissue organization is essential to better explain the mechanisms involved in the pathogenesis of many diseases, including colorectal cancer (CRC). Desmosomes are specialized structures able to mediate cell–cell contact, provide strong cell adhesion by anchoring the intermediate filament network to the plasma membrane, and mechanically integrate cells within tissues [3,4]. Physiologically, adhesive junctions confer resilience to tissues to resist mechanical stress [5]. Any alteration in the expression of desmosomal components could have an impact on the development of CRC [6]. Indeed, a dysregulation of the desmosome complex reduces the strength of cell adhesion, as well as modifies the intracellular signal transduction pathways leading to the onset and progression of CRC [7].

The desmosome plaque is constituted by three major protein families: the cadherins, the plakins, and the armadillo-repeat [8]. Desmosomal cadherins connect adjacent cells via their extracellular domains and, on the cytoplasmic side, interact with the plakin and armadillo proteins to anchor cells to the intermediate filaments (IF) keratin network. Some components of these protein families have been reported to be involved in several signaling processes [4,9,10].

Pinin (PNN/DRS/memA) is a crucial protein in stabilizing the desmosome–IF complex. PNN is a 140 kDa protein that was found not to be integral to the desmosome but strictly associated only with the mature form. In mature desmosomes, PNN interacts with the keratins of the cytoskeleton, reinforcing the strength of the IF–desmosome connection. In this way, PNN finely organizes the keratin fibers into perpendicular bundles and stabilizes the epithelial cell to cell adhesion [11,12,13]. The important activity of PNN as a putative linker protein between the IF and the desmosome plaque has been confirmed by demonstrating the direct interaction of cytokeratin-8 (KRT8) and cytokeratin-19 (KRT19) with the amino end domain of PNN [14].

Further evidence revealed that PNN is involved in multiple cellular activities since it regulates the expression of different genes at a transcriptional level and coordinates the selective splicing of pre-mRNA and lncRNA [11,15,16,17,18]. Hence, PNN may influence not only the establishment of desmosome junctions, but also pivotal processes triggered during development, tissue remodeling, and tumor progression.

MicroRNAs (miRNAs) are a class of small non-coding RNAs (20–23 nt) that are essential in the post-transcriptional regulation of gene expression. miRNAs control a broad range of biological processes such as embryonic development, cell differentiation, apoptosis, cell growth, and metabolic activity [19]. Alterations in miRNA expression lead to several pathological conditions [20,21,22,23,24]. In the past decades, many miRNAs were found to be deregulated and involved in CRC development and progression [25,26,27,28].

Much evidence has highlighted the positive effect of miRNA-based therapy on tight and adherens junction expression, although the involvement of miRNAs in CRC onset and progression via the desmosome complex is still not clearly understood. In our previous studies, we demonstrated that miR-195-5p was able to regulate a key component of desmosomes named JUP or γ-catenin as well as some desmosome cadherins (DSG2 and DSC2) [25,26]. Moreover, we showed that the increase of miR-195-5p strongly reduced tumor growth and progression through in vitro and in vivo CRC models [25,26].

In the present work, we aimed to study other mechanisms mediated by miR-195-5p in the regulation of mature desmosomes via PNN emphasizing the important role of adhesive junctions in CRC development and progression.

## 2. Results

### 2.1. PNN as a Target of miR-195-5p

In our previous works [25,26], we reported miR-195-5p as a regulator of desmosome junctions in CRC. To study further molecular targets related to the adhesive junction complex, we performed a bioinformatic analysis to predict other potential miR-195-5p target genes. Among several putative targets, we found PNN, a gene that encodes for a protein implicated in mature desmosome formation (Table S1).

### 2.2. PNN Expression in CRC Patients

**Figure 1:**
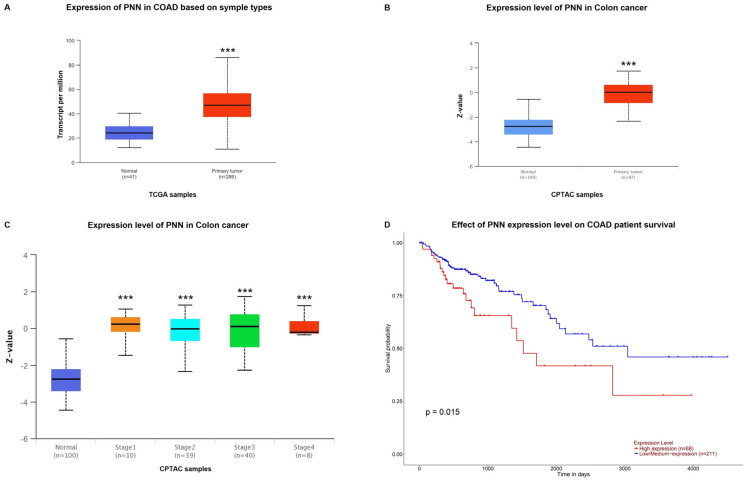
PNN as a predictive and prognostic biomarker of CRC. Analysis performed on the UALCAN tool revealed a deregulation of PNN mRNA (**A**) and protein (**B**) expression between colon adenocarcinoma (COAD) samples and adjacent normal mucosa. (**C**) Expression of PNN in the COAD dataset based on cancer stages. (**D**) Kaplan–Meier curves that represent the survival of patients with CRC included in the COAD dataset. *** *p* < 0.0001.

In order to assess whether PNN was deregulated in CRC patients, we performed a bioinformatic analysis exploiting the UALCAN tool, a web resource for analyzing cancer OMICS data from TCGA. This investigation highlighted an aberrant expression of PNN at the mRNA and protein levels in CRC tissues (*p* < 0.0001; Figure 1A,B). In addition, we evaluated PNN expression at each colon cancer stage and the effect of PNN expression levels on patient survival. Remarkably, as shown in Figure 1C, PNN expression was found to be deregulated from stage 1 of CRC (*p* < 0.001), suggesting that PNN could be a good candidate biomarker for the diagnosis and prognosis of the disease, especially in its early stages. Interestingly, PNN protein levels across the different stages of the disease remained quite similar. Figure 1D underlines that PNN expression was significantly correlated with the overall survival of CRC patients, suggesting that high expression is an unfavorable marker of CRC (*p* < 0.05).

**Figure 2:**
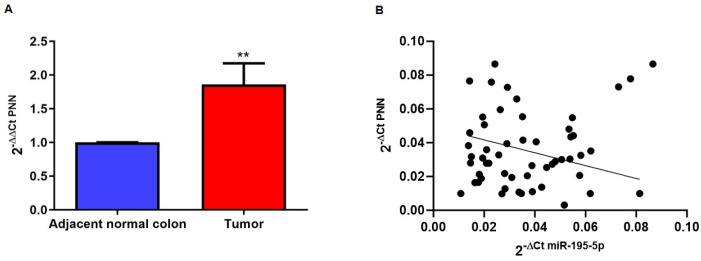
PNN expression in CRC patients (n = 30). (**A**) qPCR performed on formalin-fixed and paraffin-embedded tissue blocks of tumor and adjacent normal colon tissues. Our data showed that PNN levels were significantly increased in CRC tissue compared to normal adjacent samples. ** *p* < 0.001. (**B**) Pearson correlation analysis in all samples revealed a negative correlation between miR-195-5p and PNN expression suggesting that PNN may be regulated by miR-195-5p. r = −0.2614, *p* < 0.05.

To validate these data, we analyzed PNN expression in the tissue from a cohort of 30 CRC patients collected in our Institute. The RNA of tumoral sections and of normal adjacent samples was extracted and the qPCR amplifications revealed an upregulation of PNN mRNA in CRC tissue compared to adjacent normal colon tissue (*p* < 0.05; Figure 2A). Furthermore, we have correlated PNN expression with the miR-195-5p levels that were previously analyzed on the same tissues in our recent work [7]. As shown in Figure 2B, their expression was inversely correlated, indicating a possible regulation of PNN by miR-195-5p.

**Figure 3:**
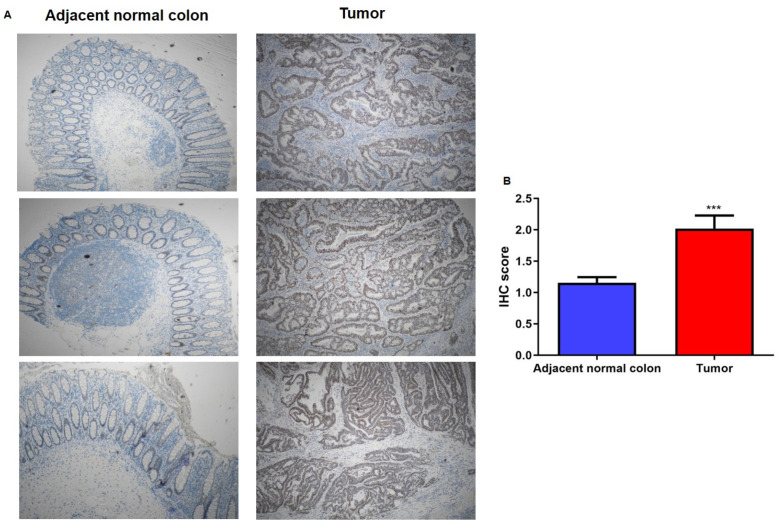
PNN protein expression in CRC and adjacent normal colon tissues. (**A**) Representative images of PNN immunoreactivity highlighted a strongly positive staining of PNN in tumor section compared to healthy control samples. Original magnification, ×4. (**B**) IHC score indicated and quantified PNN expression levels in the colonic epithelium (*** *p* < 0.0001).

Using the tissue specimens from the same cohort of CRC patients, we also evaluated PNN protein expression by IHC analysis. As highlighted in Figure 3, PNN expression was strongly upregulated in CRC tissue compared to the corresponding peritumoral sample (Figure 3A) and these data were also confirmed by defining an immunoreactivity score from 0 to 3 (absent to strong) based on PNN staining intensity (*p* < 0.0001; Figure 3B).

### 2.3. miR-195-5p Is an Effective Regulator of PNN Expression

**Figure 4:**
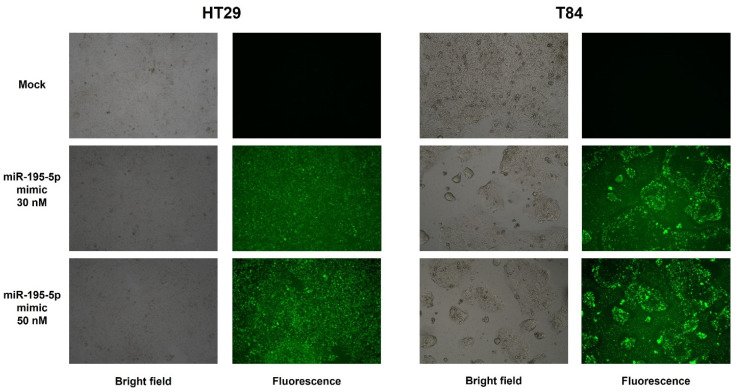
Transfection efficiencies of HT29 and T84 cell lines. Cells were transiently transfected with FAM-labeled miR-195-5p mimic at 30 nM and 50 nM concentrations. Bright field (**left**) and fluorescence (**right**) representative images for the mock condition and transfected conditions were acquired at 20× magnification.

To functionally validate the putative target prediction, we transiently transfected cells with molecules of miR-195-5p mimic at concentrations of 30 nM and 50 nM. We first evaluated the transfection efficiency of HT29 and T84 human epithelial cells and our results showed an increase of intracellular levels of miR-195-5p in FAM-labeled miR-195-5p mimic-transfected conditions (Figure 4). The transfection efficiencies at 30 nM and 50 nM in HT29 were 70% and 85% while in T84 they were about 60% and 76%.

**Figure 5:**
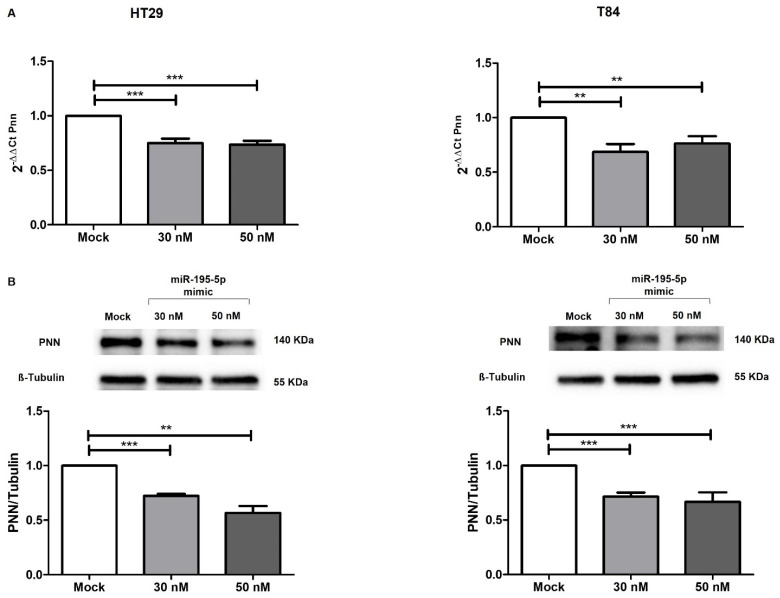
PNN expression after miR-195-5p mimic transfection. (**A**) PNN mRNA expression was evaluated after miR-195-5p mimic transfection in HT-29 (**left**) and T84 (**right**) human cell lines. The increased intracellular amount of miR-195-5p (at 30 nM and 50 nM concentrations) induced an effective reduction of PNN in both cell lines. Expression data were normalized to those of the housekeeping gene Gapdh and are representative of four independent experiments (mean ± SEM). ** *p* < 0.001; *** *p* < 0.0001. (**B**) PNN protein expression was analyzed by Western blotting. The raise of miR-195-5p at 30 nM and 50 nM concentrations led to a significant decrease of PNN levels in HT29 and T84 cell lines. PNN values were obtained by dividing the normalized transfected sample values by the normalized mock–control sample values. Data were normalized to the values of housekeeping β-tubulin. ** *p* < 0.001; *** *p* < 0.0001.

Enhancing the mature form of miR-195-5p in HT-29 and T84 cell lines, we aimed to probe whether miR-195-5p controlled PNN expression. Data obtained by real-time PCR demonstrated that the PNN mRNA expression level in transfected conditions was significantly decreased in all cell lines (*p* < 0.001; Figure 5A).

Western blot analysis confirmed the results obtained by qPCR, highlighting that the gain of function of miR-195-5p was able to significantly reduce the levels of PNN protein in transfected cells (*p* < 0.001; *p* < 0.0001 Figure 5B).

### 2.4. miR-195-5p Indirectly Modulates the Expression of Cytokeratin-8 and Cytokeratin-19

**Figure 6:**
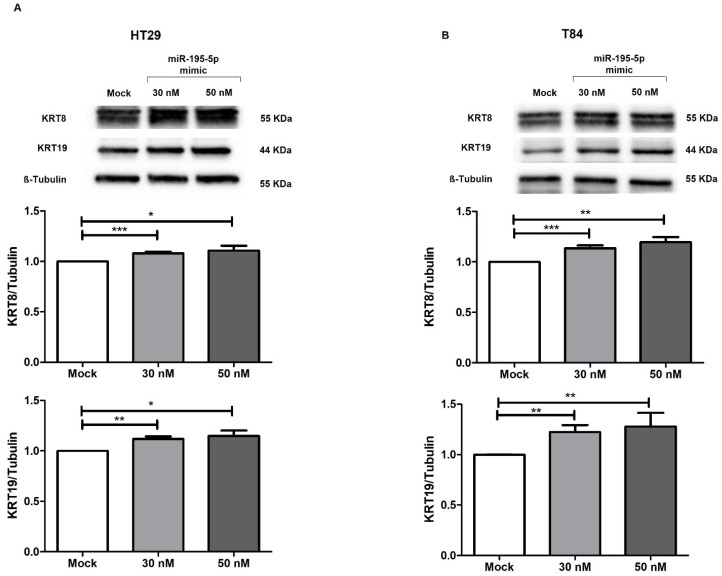
miR-195-5p effect on KRT8 and KRT19 protein expression. The gain of function of miR-195-5p in HT29 (**A**) and T84 (**B**) regulated PNN expression that, in turn, modulated KRT8 and KRT19 protein levels. Our findings showed an increase of KRT8 and KRT19 in transfected conditions compared to mock control. Data are representative of four independent experiments (n = 4; mean ± SEM) and were normalized to β-tubulin housekeeping values. * *p* < 0.01, ** *p* < 0.001, *** *p* < 0.0001.

To characterize the involvement of miR-195-5p in the regulation of desmosome structure, we evaluated the expression of key keratins that closely interact with PNN. Prediction of the interacting proteins performed using the Genemania database indicated a physical interaction between PNN and KRT8 and KRT19 (Figure S1). This binding could reorganize the keratin fibers and enhance the strength of desmosome junctions. Indeed, the increase of intracellular levels of miR-195-5p at 30 nM and 50 nM concentrations led to an indirect upregulation of KRT8 and KRT19 through targeting PNN expression (*p* < 0.05; *p* < 0.001; *p* < 0.0001 Figure 6).

To further evaluate the effect of miR-195-5p on PNN, KRT8, and KRT19 expression, we performed the immunofluorescence assay. As highlighted in Figures S2 and S3, the immunostaining of HT29 and T84 monolayers confirm the data obtained by Western blot. Indeed, the miR-195-5p mimic transfection at 30 nM and 50 nM was able to significantly reduce PNN expression as well as indirectly modulate the expression of KRT8 and KRT19 in both cell lines.

### 2.5. PNN Determination after miR-195-5p Mimic Treatment in a Mouse Model of AOM/DSS-Induced CRC

In our recent work, we have characterized the in vivo therapeutic effect of miR-195-5p, employing an azoxymethane (AOM)/dextran sodium sulfate (DSS)-induced CRC mouse model [26]. Briefly, male C57BL/6 mice (7 weeks old; 19–25 g) were divided into a vehicle group and miR-195-5p-treated group and subjected to cycles of AOM and DSS administration, followed by distilled water for 15 days. For the miR-195-5p treated group, the injections were administered twice a week until sacrifice.

**Figure 7:**
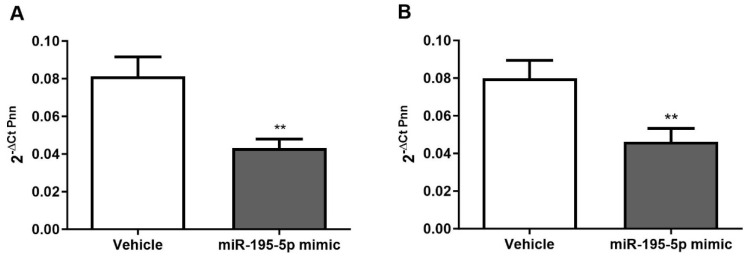
In vivo effectiveness of miR-195-5p on PNN expression after its administration to AOM/DSS-treated mice (n = 14 mice/group). PNN expression in the medial (**A**) and distal colons (**B**) of miR-195-5p-treated and untreated mice evaluated by real-time PCR underlined a substantial decrease of Pnn levels after miR-195-5p administration. Expression data were normalized to those of the housekeeping gene Gapdh and are represented as mean ± SEM. ** *p* < 0.001.

To further elucidate the positive role of miR-195-5p in inhibiting AOM/DSS-induced tumorigenesis, we evaluated PNN expression levels in the medial and distal colons of vehicle and miR-195-5p-treated mice established in our previous work [26]. The intraperitoneal administration of miR-195-5p mimic led to a remarkable decrease of PNN expression of miR-195-5p in the colon portion of treated mice compared to the control group (*p* < 0.001; Figure 7). These data are in strong accordance with our in vitro results, confirming that PNN could be a good candidate target for miR-195-5p-based therapy in CRC.

## 3. Discussion

Cell junctions deeply characterize epithelial morphology and function, ensuring the integrity and the tensile strength of tissues [5]. These characteristics of epithelial cells are guaranteed by several specialized structures including desmosomes that connect cells to intermediate filaments or adherens junctions and tight junctions which are strictly linked to cellular microfilaments [29,30]. The cell connections to the cytoskeletal network provide a stable cell–cell adhesion but also ensure the changes in morphology typical of epithelial cells [31,32,33]. Desmosomes play an essential role in tissue integrity and homeostasis by forming robust extracellular bonds that connect the cell surface to the cytoskeleton [34]. In addition, desmosome structural properties facilitate cell–cell communication and the processing of context-dependent signals emerging as mediators of cell signaling [35].

Among several proteins involved in the establishment of adhesive junctions, PNN is dynamically recruited to pre-formed desmosomes but it is absent from nascent desmosomes [36]. Indeed, PNN is not integral to desmosome assembly, but it is correlated to the organization and stabilization of keratin intermediate filaments binding the cytoskeleton network to the lateral surface of the plasma membrane [14].

Another function of PNN was reported to be associated to the regulation of gene expression or pre-mRNA alternative splicing interacting with transcription regulators or members of the RNA recognition motif family, respectively [37,38,39]. As a consequence, an alteration in desmosome composition or components was associated with tumor progression and the deregulation of PNN was related to embryonic development, cellular differentiation, tumorigenesis, and metastasis [40,41,42,43,44,45]. Indeed, PNN was reported to have proliferation- and motility-promoting effects, activating CREB via the PI3K/AKT and ERK/MAPK pathways [11]. Interestingly, the deregulation of PNN was related to CRC tumor progression in vitro and in vivo, activating the EGFR/ERK pathway [36]. Furthermore, starting from an RNA seq transcriptomic approach, PNN was also identified and validated as a potentially predictive biomarker of drug response in CRC [46,47].

In the last few years, miRNA properties as gene regulators at post-transcriptional levels and their implication in the pathogenesis of several diseases, including CRC, have gained broad interest among researchers for therapeutic purposes [48,49]. Some findings highlighted the effects of miRNAs in CRC progression, regulating tight junction and adherence junction expression [50,51]. In this way, it has been demonstrated that the overexpression of miR-21 downregulated both Claudin-1 and E-cadherin expression promoting the epithelial-to-mesenchymal transition (EMT) and the invasiveness of colorectal adenocarcinoma cells [52]. Zhou and co-workers showed that miR-214-3p induces the EMT process of CRC cell lines, acting on the expression of E-cadherin and ZO-1 [53].

To date, the correlation between miRNAs and desmosome components, especially PNN, in CRC development is still poorly investigated. In our recent previous studies, we have characterized the role of miR-195-5p in the regulation of desmosome junctions, finding that miR-195-5p was able to target the expression of JUP (γ-catenin) and consequently to modulate the levels of desmosome cadherins DSG2 and DSC2 as well as the expression of the key effectors of Wnt signaling NLK, LEF1, and Cyclin D1 [25,26]. In addition, JUP regulation by miR-195-5p had an unequivocal effect on CRC progression, inhibiting the proliferation, viability, migration, and invasion of colonic epithelial cells as well reducing the in vivo numbers and sizes of tumors in AOM/DSS-treated mice [25,26].

In the present study, with the aim to investigate further mechanisms related to the desmosome complex through a bioinformatic approach, we identified PNN, a gene that encodes for a mature desmosome component, as a miR-195-5p putative target. Here, using a public data repository, we found that PNN expression was overexpressed in colon cancer tissues compared to normal adjacent mucosa at both the mRNA and protein levels. Moreover, the abnormal expression of PNN was identified from stage 1 of the disease and its expression was significantly correlated with the overall survival of CRC patients, suggesting that PNN could be a potential biomarker and a good candidate target for a miR-195-5p based therapy.

To confirm these data, we evaluated PNN expression in tissue specimens collected in our Institute that included the tumoral section and adjacent normal portion from CRC patients, confirming the upregulation of PNN in pathological conditions.

We have also biologically investigated the effect of miR-195-5p on PNN expression, revealing that the in vitro gain of function of miR-195-5p led to a significant decrease of PNN mRNA and protein levels. As a consequence of PNN regulation by miR-195-5p, the expression of KRT8 and KRT19, closely connected to PNN, was affected. Since the physical interaction of KRT8 and with PNN was previously demonstrated [14], we supposed that the effect of miR-195-5p on PNN expression may lead to an indirect modulation of also these cytokeratins. Indeed, we highlighted an increased expression of KRT8 and KRT19 that may reorganize the cell anchoring and enhance the strength of desmosome junctions.

Finally, we observed that the in vivo administration of miR-195-5p in AOM/DSS-treated mice led to a strong downregulation of PNN expression in the medial and distal colons of treated mice compared to the control group. These data are in strongly accordance with the results obtained in our previous work, where we demonstrated the in vivo positive effect of miR-195-5p on tumor growth.

Altogether, our results elucidated another mechanism controlled by miR-195-5p in the regulation of the desmosome structure and the effective involvement of this miRNA in the development of CRC. Indeed, we have identified a novel miR-195-5p/PNN axis that is able to affect the desmosome complex. However, this study has a limitation; our data are preliminary since demonstrate the regulation of PNN expression by miR-195-5p in CRC. Future studies will be needed to biologically characterize the involvement of PNN in CRC progression.

## 4. Materials and Methods

### 4.1. Human Tissues

Formalin-fixed and paraffin-embedded (FFPE) human tissues used for the molecular analysis were derived from thirty patients affected by CRC that were retrospectively enrolled at the National Institute of Gastroenterology “S. de Bellis” Castellana Grotte, Bari, Italy. The following are the inclusion criteria for this study: (1) patients older than 18 years; (2) patients with confirmed colorectal adenocarcinoma by histopathological examination; (3) gene mutation status wild-type; (4) patients naïve to neoadjuvant chemotherapy. The following are the exclusion criteria: (1) patients with more than one primary tumor location; (2) patients with lower rectum cancer that are undergoing treatment before chirurgical resection. For each patient, we collected tumoral and adjacent normal samples. Tissues were sectioned, stained with hematoxylin and eosin, and analyzed by a pathologist. All patients signed a written informed consent and all experiments were carried out according to the principles of the Declaration of Helsinki and approved by the local institutional ethics review board (Istituto Tumori Giovanni Paolo II, Bari, Italy, n° 379/2020 of 16 September 2020).

### 4.2. RNA Extraction and Real-Time PCR of FFPE Tissue

FFPE specimens were treated with a Deparaffinization Solution (Qiagen, Hilden, Germany) and then the total RNA, including miRNAs, was extracted with the miRNeasy FFPE kit (Qiagen, Hilden, Germany) according to the manufacturer’s instructions.

Total RNA was retrotranscribed using SuperScript™ VILO™ MasterMix (Thermo Fisher Scientific, Bremen, Germany) and the qPCR amplification reactions were elicited on a CFX96 System (Biorad Laboratories, Hercules, CA, USA) using the SsoAdvanced Universal SYBR Green Supermix (BioRad Laboratories, Hercules, CA, USA) and the QuantiTect Primer Assay for PNN and GAPDH (Qiagen, Hilden, Germany). Data of comparative qPCR was obtained in triplicates and GAPDH gene amplification was used as a reference standard to normalize the relative expression of PNN. The relative expression was calculated using the 2^−ΔΔCt^ formula.

### 4.3. Immunohistochemistry (IHC)

Sixty FFPE tissues of CRC patients were used for IHC analysis. Three µm sections were cut and mounted on Apex Bond IHC slides (Leica Biosystems, Buffalo Grove, IL, USA). IHC staining procedures were performed on the BOND III automated immunostainer (Leica Biosystems, Buffalo Grove, IL, USA), from deparaffinization to counterstaining with hematoxylin. To assess PNN staining, the anti-PNN primary antibody (ab244250, Abcam, Cambridge, UK; 1:200 dilution) was used. Antigen retrieval was performed with BOND Epitope Retrieval Solution 2 (Leica Biosystems, Buffalo Grove, IL, USA) using citrate buffer pH 6. The Bond Polymer Refine Detection Kit (Leica Biosystems, Buffalo Grove, IL, USA) was used as a visualization and chromogen reagent according to the manufacturer’s protocol. The samples were considered negative if the number of stained cells was less than 5%.

For quantification of the positive signal, PNN expression was scored as follows: 0, (no staining) negative; 1, weak expression; 2, moderate expression; and 3, strong expression.

### 4.4. Cell Culture and In Vitro Transfection

Human colonic epithelial cells, HT29 and T84, were purchased from ATCC (American Type Culture Collection, Manassas, VA, USA). HT29 were maintained in Dulbecco’s Modified Eagle Medium (DMEM, Thermo Fisher Scientific, Waltham, MA, USA) supplemented with 10% heat-inactivated Fetal Bovine Serum (FBS, Thermo Fisher Scientific, Waltham, MA, USA), 1% 10,000 µg/mL streptomycin and 10,000 U/mL penicillin (Thermo Fisher Scientific, Waltham, MA, USA), 1% 1M HEPES (Sigma-Aldrich, St. Louis, MO, USA), and 1% 100 mM sodium pyruvate (Sigma-Aldrich, St. Louis, MO, USA). T84 were cultured in Dulbecco’s Modified Eagle Medium: Nutrient Mixture F-12 (DMEM:F12, Thermo Fisher Scientific, Waltham, MA, USA) supplemented with 10% FBS (Thermo Fisher Scientific, Waltham, MA, USA) and 1% streptomycin/penicillin (Thermo Fisher Scientific, Waltham, MA, USA). All cell lines were maintained at 37 °C in a 5% of CO_2_.

For RNA extraction, all cell lines were plated into 12-well plates at a density of 1.5 × 10^5^ cells/well, while for protein lysis HT29 and T84 were seeded into 6-well plates at a density of 2 × 10^5^ and 4.5 × 10^5^ cells/well respectively. At confluence, the cells were transiently transfected with synthetic molecules of miR-195-5p mimic at concentrations of 30 and 50 nM (Life Technologies, Carlsbad, CA, USA) using TransIT-TKO Transfection Reagent (Mirus Bio LLC, Madison, WI, USA) according to the manufacturer’s protocol. In each experiment, a mock control consisting of cells transfected with TKO transfection reagent alone was used.

### 4.5. RNA Extraction and Real-Time PCR

Total RNA was extracted from mice tissue and from cell cultures using TRIzol reagent (Invitrogen by Thermo Fisher scientific, Waltham, MA, USA), eluted in ribonuclease-free water, and analyzed with the NanoDrop ND-2000 Spectrophotometer (Thermo Fisher Scientific, Waltham, MA, USA) to determine its concentrations.

RNA was then reverse transcribed using the iScript Reverse Transcription Supermix (BioRad Laboratories, CA, USA) following the manufacturer’s recommendations. Quantitative real-time PCR was performed on a CFX96 System (Biorad Laboratories, Hercules, CA, USA) using the SsoAdvanced Universal SYBR Green Supermix (BioRad Laboratories, Hercules, CA, USA) and the QuantiTect Primer Assay for PNN and GAPDH (Qiagen, Hilden, Germany).

The expression levels of the target gene and housekeeping gene were determined from four independent experiments. GAPDH gene amplification was used as reference standard to normalize the relative expression of PNN. The relative expression of genes was calculated using the 2^−ΔΔCt^ formula.

### 4.6. Protein Isolation and Western Blot Analysis

For protein extraction, HT29 and T84 cell lines were lysed seventy-two hours after transfection using T-PER Tissue Protein Extraction Reagent (Thermo Fisher Scientific, Waltham, MA, USA) supplemented with cocktail proteinase inhibitors (Sigma-Aldrich, St. Louis, MO, USA). Using the Bradford colorimetric assay (Bio-Rad Laboratories, Richmond, CA, USA), the total protein concentration was determined. For each protein lysate, 60 μg of total proteins were separated in 4–20% Mini-PROTEAN TGX Stain-Free Protein Gels (Biorad Laboratories, Hercules, CA, USA) and transferred onto Trans-Blot Turbo Mini 0.2 µm PVDF membranes (Biorad Laboratories, Hercules, CA, USA). For protein detection, PVDF membranes were incubated in iBind automated Western Systems (Thermo Fisher Scientific) and the immunoblot band densities were detected with a Chemidoc System (Biorad Laboratories, Hercules, CA, USA). The images were analyzed with Image Lab Software version 5.2.1 (Biorad Laboratories, Hercules, CA, USA), and quantified by ImageJ Software version 1.54d.

For protein detection, primary antibodies included rabbit anti-PNN (ab244250, Abcam, Cambridge, UK; 1:500 dilution), mouse anti-KRT8 (ab9023; Abcam, Cambridge, UK; 1:5000 dilution), mouse anti-KRT19 (ab7754, Abcam, Cambridge, UK; 1:5000 dilution), and mouse monoclonal β-tubulin (sc-166729, Santa Cruz Biotechnology, Inc., Heidelberg, Germany; dilution 1:1000). Secondary antibodies goat anti-mouse IgG-(H+L)-HRP conjugate (170-6516, Biorad Laboratories, Hercules, CA, USA; dilution 1:500) and goat anti-rabbit IgG-(H+L)-HRP conjugate (31466, Invitrogen, Carlsbad, CA, USA; dilution 1:2500) were used.

### 4.7. Immunofluorescence

HT-29 and T84 cells were seeded in Lab-Tek Chamber Slides and transfected at confluence. The monolayers were fixed with PFA 4% for 10 min at 4 °C, washed twice with PBS, and then permeabilized with Triton-X 0.1% in PBS for 5 min at room temperature. Subsequently, cells were blocked in PBS + BSA 3% for 1.5 h at room temperature and incubated in primary antibody rabbit PNN (ab244250, Abcam, Cambridge, UK; 1:100 dilution), mouse anti-KRT8 (ab9023; Abcam, Cambridge, 352 UK; 1:100 dilution), or mouse anti-KRT19 (ab7754, Abcam, Cambridge, UK; 1:100 dilution) diluted in PBS + BSA 3% for 3 h. After washing with PBS, they were incubated with secondary antibody chicken anti-rabbit IgG (H+L) Alexa Fluor 594 (A-21442, Invitrogen, dilution 1:400), and chicken anti-mouse IgG (H+L) Alexa Fluor 488 (A-21200, Invitrogen, dilution 1:400) diluted in PBS + BSA 3% for 1 h. ProLong Gold Antifade Mountant with DAPI (Thermo Fisher Scientific) was applied to each sample, mounted with a glass cover slip. Images were assessed using a fluorescence microscope (Eclipse Ti2, Nikon Inc., Melville, NY, USA) using filters for DAPI, FITC, and RFP/TRITC.

### 4.8. Bioinformatic and Statistical Analysis

UALCAN tools (https://ualcan.path.uab.edu/; accessed on 27 March 2023) [54] were used to analyze PNN gene and protein expression in CRC tissue compared to normal colon as well as its expression in the different stages of CRC. Furthermore, it was used also to determine the correlation between PNN expression and patient survival. To predict the specific interaction of PNN networks, the GeneMANIA resource (https://genemania.org/; accessed on 10 April 2023) [55] was examined.

Putative miR-195-5p gene targets were predicted by means of the miRabel (http://bioinfo.univ-rouen.fr/mirabel/; accessed on 13 March 2023) [56], Tarbase (https://dianalab.e-ce.uth.gr/html/diana/web/index.php?r=tarbasev8/index; accessed on 14 March 2023) [57], miRNet (https://www.mirnet.ca; accessed on 14 March 2023) [58], TargetMiner (https://www.isical.ac.in/~bioinfo_miu/targetminer20.htm; accessed on 15 March 2023) [59], and miRGator (http://mirgator.kobic.re.kr/; accessed on 15 March 2023) [60] algorithms.

Data were analyzed using GraphPad Prism software version 10.0.2 and expressed as the mean ± SEM. A Pearson test was applied to study miRNA and mRNA correlation. Statistical significance of data resulting from different conditions was examined with two-tailed Student’s *t* test. Data derived from at least three independent experiments were considered statistically significant at *p* < 0.05.

## 5. Conclusions

In conclusion, in this paper, we have demonstrated that PNN, a key component of mature desmosomes, was significantly overexpressed in CRC patients. miR-195-5p was able to reverse the aberrant expression of PNN in CRC cell lines and to strongly reduce PNN expression levels in the medial and distal colon of AOM/DSS-treated mice. These findings provide further evidence of the clinical potential of miR-195-5p in the regulation of adhesive junctions and in CRC progression.

## Data Availability

Data are within the article and Supplementary Materials.

## Supplementary Materials

The following supporting information can be downloaded at: https://www.mdpi.com/article/10.3390/ijms25115980/s1.
