# Supplementary figures and images for: Identification of a Novel miR-195-5p/PNN Axis in Colorectal Cancer
Source: Int J Mol Sci. 2024 May 30;25(11):5980. doi: 10.3390/ijms25115980 (PMC11172886; doi:10.3390/ijms25115980)

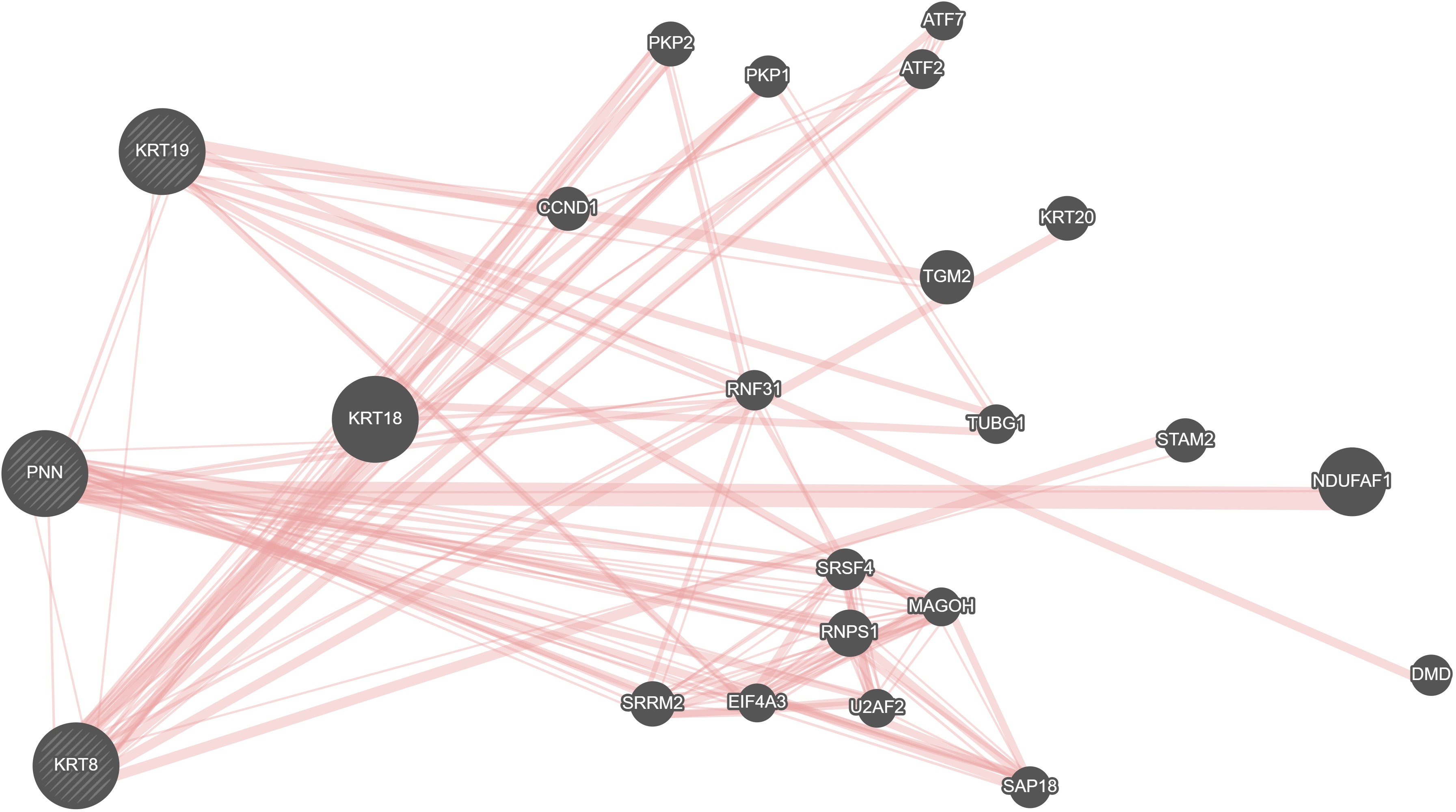

Supplement: Supplementary file 1 [file ijms-25-05980-s001.zip › Figure S1.jpg]

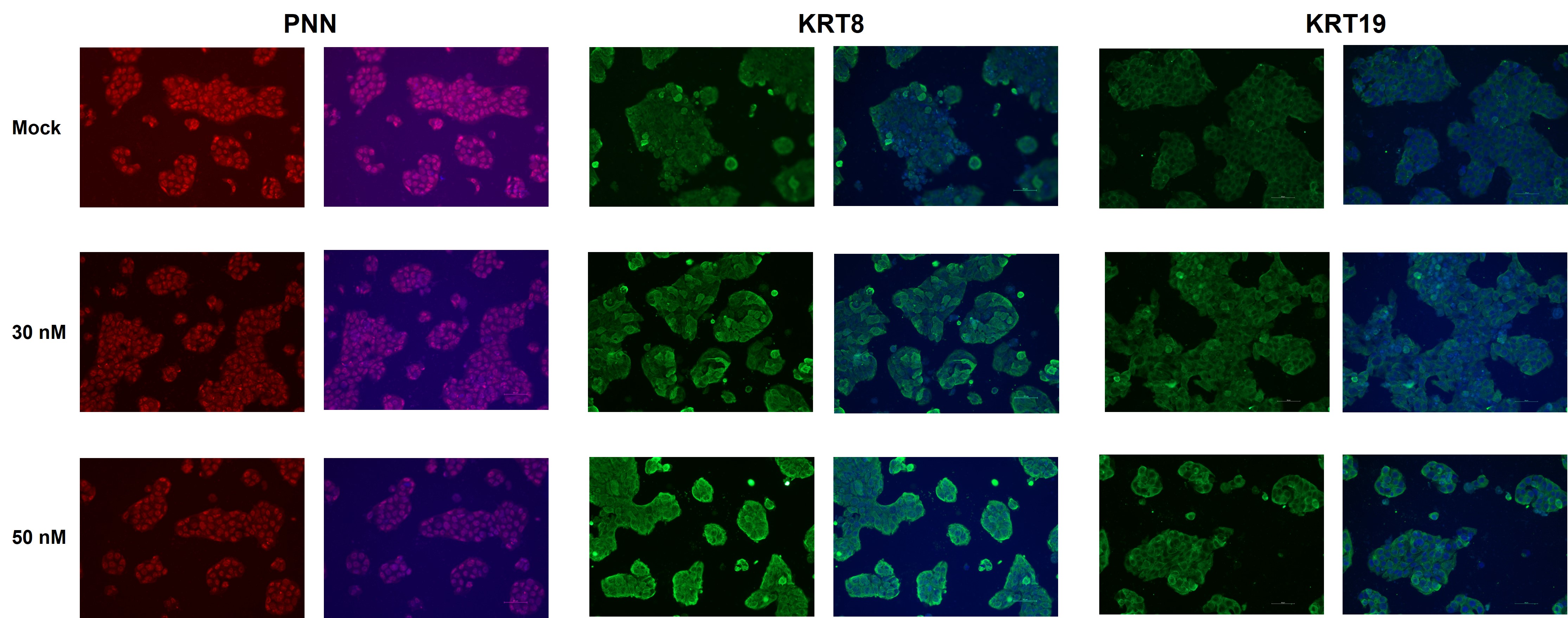

Supplement: Supplementary file 1 [file ijms-25-05980-s001.zip › Figure S2.jpg]

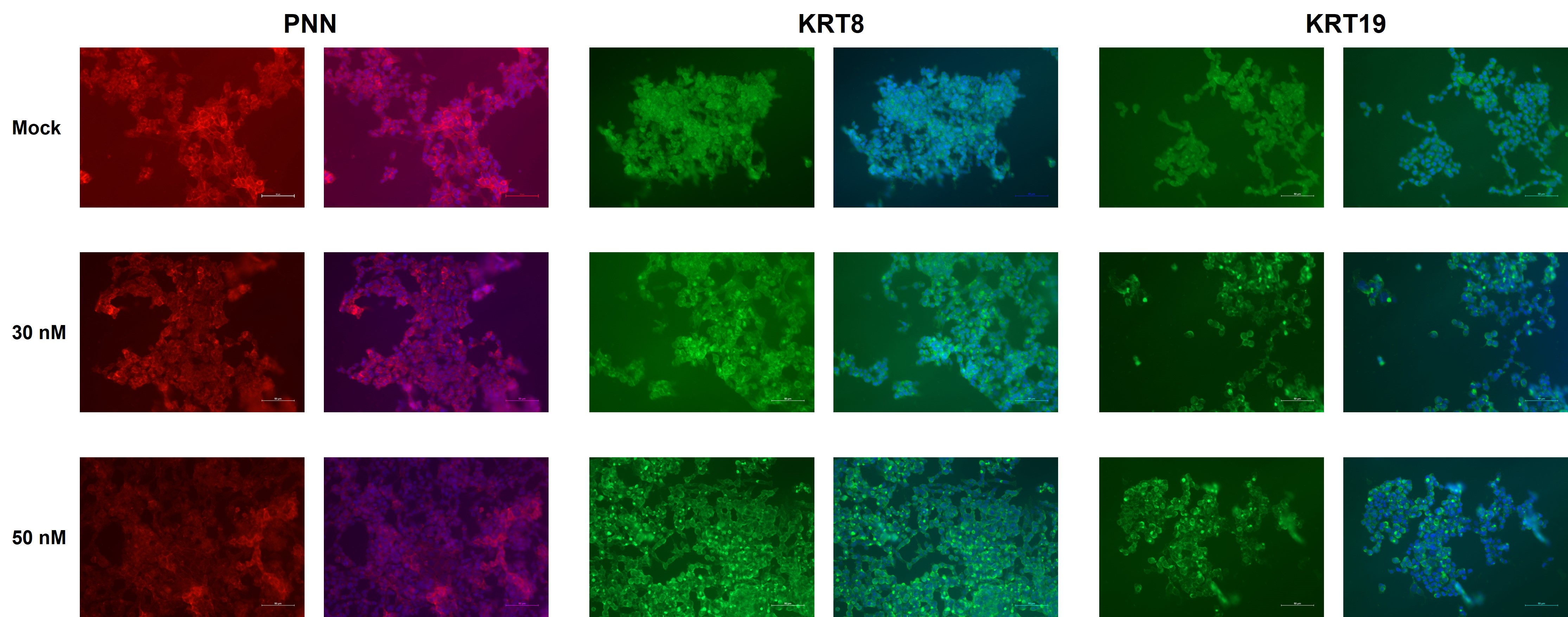

Supplement: Supplementary file 1 [file ijms-25-05980-s001.zip › Figure S3.jpg]
